# Identify Early and Involve Everyone: Interdisciplinary Comprehensive Care Pathway Developed for Inpatient Management and Transitions of Care for Heart Failure Patients Reported Using SQUIRE 2.0 Guidelines

**DOI:** 10.7759/cureus.21123

**Published:** 2022-01-11

**Authors:** Rishi Thaker, Kevin Pink, Sita Garapati, Donna Zarandi, Purvi Shah, Kumudha Ramasubbu, Parag Mehta

**Affiliations:** 1 Internal Medicine, New York-Presbyterian Brooklyn Methodist Hospital, Brooklyn, USA; 2 Cardiology, New York-Presbyterian Brooklyn Methodist Hospital, Brooklyn, USA; 3 Internal Medicine, NewYork-Presbyterian Brooklyn Methodist Hospital, Brooklyn, USA

**Keywords:** clinical care delivery and management, hospital readmission, care pathways, health care transition, decompensated heart failure

## Abstract

Introduction

Heart failure accounts for 1-2% of overall healthcare costs. While the link between re-hospitalization and mortality is unclear, care pathways that standardize inpatient management and establish outpatient follow-up improve patient outcomes and reduce morbidity.

Aim

To implement a comprehensive interdisciplinary care pathway for heart failure patients with the goal of optimizing inpatient management and improving transitions of care.

Methods

To address this clinical need, New York-Presbyterian Brooklyn Methodist Hospital (NYP-BMH) identified resources needed to optimize patient care, developed an inpatient admission order set (so-called “power plan”), and implemented a multidisciplinary clinical care pathway. The Plan-Do-Study-Act cycle addressed the implementation obstacles. Interdisciplinary rounds guided day-to-day management and addressed barriers. Our team developed a sustainable care pathway, and measured the utilization of pharmacy, nutrition, physical therapy, case management, and social work resources; outpatient appointments were made prior to discharge. We used the Standards for Quality Improvement Reporting Excellence (SQUIRE) 2.0 guidelines to guide our planning and evaluation of this quality improvement initiative.

Results

Our intervention markedly increased the number of heart failure hospitalizations that were identified on admission, and the use of pharmacy/nutrition services was greater after the intervention. The utilization of our “power plan” promoted adherence to a series of evidence-based best practices, but these measures had no significant impact on readmissions as a whole. The involvement of the case management support team increased outpatient appointments made for patients prior to discharge and aided in the transition of care from inpatient to outpatient management.

Conclusion

The management of heart failure patients starts in the hospital and continues in the community. Patients who are treated in a standardized dedicated care pathway have reduced morbidity and better outcomes. Identifying these patients early, involving a comprehensive team, and transitioning their care to the outpatient setting improves the quality of care in these patients.

## Introduction

Heart failure admissions account for 1-2% of overall health care costs in the United States, over $30 billion each year [1], and domestic prevalence exceeds 6.2 million patients [2]. Heart failure readmissions are more common than readmissions for pneumonia or myocardial infarction [3]. Yet, recent evidence suggests that reducing heart failure readmissions may paradoxically be associated with increased mortality because sicker patients benefit from frequent contact with the healthcare system [4,5].

Patients with heart failure are at higher risk of admission to the hospital for any cause, and more than 86% of readmissions may be due to other morbidities besides heart failure [6]. In fact, the minority of heart failure admissions are considered overtly preventable, while the majority are considered multifactorial [7]. A critical component of management for heart failure patients involves establishing and implementing a treatment plan that optimizes the inpatient care and outpatient transition. Suboptimal discharges neglect important facets of the care continuum and ultimately fail to reduce patient morbidity. Without nutritional education, the patient is more likely to suffer from heart failure symptoms [8], without pharmacist medication reconciliation the patient is at higher risk of mortality and hospitalization [9,10], and without a primary care appointment at the time of discharge, the patient is less likely to come for a follow-up [11].

In-hospital and post-hospital care pathways are emerging concepts in healthcare delivery. In addition to a multidisciplinary approach, these pathways are most effective with inpatient education, appropriate discharge planning, and dedicated care pathways among a team of specialized nurses and doctors [11-17]. High-quality discharge instructions delivered to the patient’s primary care doctor, and improved primary care follow-up also reduce readmission [14,18,19]. Dedicated care pathways have been investigated in stroke management, soft tissue infections, diabetes treatment, and asthma exacerbation with positive results [20-23]. A multidisciplinary approach utilizes pharmacy, case management, nursing, patient education, and medical management to optimize care [18,19,24]. The aim of this study was to collect data pre-and post-implementation of a care coordination pathway that optimized heart failure management within our hospital.

## Materials and methods

Framework

Three previous randomized trials served as the template for our care pathway initiative, which are summarized in Table 1. Common threads of these successful programs by Naylor et al. [11], Jack et al. [12], and Coleman et al. [13] involved in-hospital patient education, engagement by a multidisciplinary team, and discharge planning with education, medication reconciliation, and follow-up phone calls. Discharge planning and transition of care were the cornerstones of each intervention, and these interventions were demonstrated to work in an academic setting, community setting, and in safety-net hospitals in various cities across the United States. The outcomes of these interventions produced a reduction in hospital readmission, with a number needed-to-treat as low as seven in one study. We used the Standards for Quality Improvement Reporting Excellence (SQUIRE) 2.0 guidelines to guide our planning and evaluation of this quality improvement initiative.

**Table 1:** Framework studies for our Care Coordination initiative Naylor et al., 1994 [13]; Coleman et al., 2004 [12]; Jack et al., 2009 [14]

Author, year	Intervention	Setting; study sample	Key elements	Outcomes
Naylor et al., 1994	Comprehensive discharge planning	Academic hospital in Philadelphia; Community-dwelling elders with selected medical and surgical conditions and their caregivers	Advanced practice nurse: - Meets with patient and caregiver in the hospital - Follows every 48hr as needed - At least two follow-up telephone calls post-discharge - Available as needed for questions	The reduced readmission rate for medical patients at 6 weeks (10% vs. 23%, p<0.05)
			Intervention components: - Structured assessment of patient and caregiver needs - Comprehensive discharge planning - Patient and caregiver education - Ongoing assessment and adjustment of the plan if needed - care coordination for up to two weeks post-discharge - Interdisciplinary communication	
Coleman et al., 2004	Care Transitions Intervention	Not-for-profit health system in Colorado; Community-dwelling elders with selected medical and surgical conditions	Nurse transition coach: - Meets with the patient in the hospital - Home visits 48-72 hours after discharge - Three follow-up telephone calls	Reduced readmission rate at 30 days (8.3% vs. 11.9%, p<0.05) and 90 days (16.7% vs. 22.5%, p<0.05) in adjusted analysis
			Four pillars: - Medication self-management - Patient-owned health record - Timely outpatient follow-up - Awareness of red flags and appropriate actions to take	
Jack et al., 2009	Project Reengineering Discharge (RED)	Safety-net hospital in Boston; Adults admitted to medical teaching services	Nurse discharge advocate: - Meets with the patient throughout the hospital stay - Coordinates in-hospital discharge planning - Prepares after hospital care	Reduced hospital utilization (combined endpoint of emergency department visits and rehospitalization) at 30 days, incidence rate ratio = 0.695 (95% CI, 0.515 to 0.937)
			Intervention components: - Patient education - Schedule follow-up appointments - Pharmacist reconciliation - Review test results and outstanding tests - Organize post-discharge services - Medication reconciliation - Reconcile discharge plan with care pathways and guidelines - Discuss action plan in case of problems - Transmit discharge summary to the following provider - Assess patient understanding - After-hospital care plan [written patient education and instructions in plain language(s)] - Telephone reinforcement	

Specific aims

The purpose of our project was to optimize the management of patients during acute hospitalization and optimize the post-discharge transition of care in heart failure patients. This study was granted exempt status by New York Methodist Hospital Institutional Review Board (IRB reference no. 1495869, November 5, 2019) given that all patient data was de-identified, and patients were not randomized into different treatment arms.

Planning and drivers

An executive committee incorporated these various models of addressing heart failure readmission. For example, Jack et al. demonstrated comprehensive discharge planning, along with patient education and care coordination post-discharge were significant contributors to improved patient outcomes [14]. Additionally, patient/caregiver education and interdisciplinary communication served as important components of the care pathway. In Coleman et al.'s study, medication self-management and timely outpatient follow-up were central pillars [12]. In Jack et al.'s study, organizing post-discharge services, planning after-hospital care, transmitting discharge instructions to the outpatient provider, and ensuring medication reconciliation produced a marked reduction in healthcare utilization.

We studied our electronic medical record (EMR) data and decided to address heart failure admissions on two fronts. We called them primary and secondary drivers which are summarized in Table 2. the primary drivers of our care coordination initiative were to a) optimize inpatient management of heart failure patients and b) optimize management of post-acute care. Secondary drivers were to a) appropriately identify patients with active heart failure, b) develop an aggressive treatment “power plan” in our electronic medical record, and c) ensure adherence to one-week primary care or cardiology follow-up and medication and diet recommendations.

**Table 2:** Primary and secondary drivers of the Care Coordination initiative

Primary Drivers	Secondary Drivers
Optimize inpatient medical management	Identify patients with active heart failure
Optimize management of post-acute care	Rapid, aggressive treatment with “power-plan”
	Adherence to medication, diet, and follow-up appointment w/in 1 week

Context

Prior to the implementation of our readmission initiative, we were using our resources in a provider-dependent manner. Translating best practices from research to clinical practice can take as long as 17 years [25] and developing a dedicated care pathway for heart failure admissions was a top priority for the hospital at the outset of this project. Additionally, readmission penalties penalize under-served and safety-net hospitals financially, which makes them a target for intervention [26,27]. Rather than target readmissions, the goal of our project was to coordinate care along the inpatient-outpatient continuum and give access to heart failure patients in the acute-care setting.

Interventions

**Figure 1:**
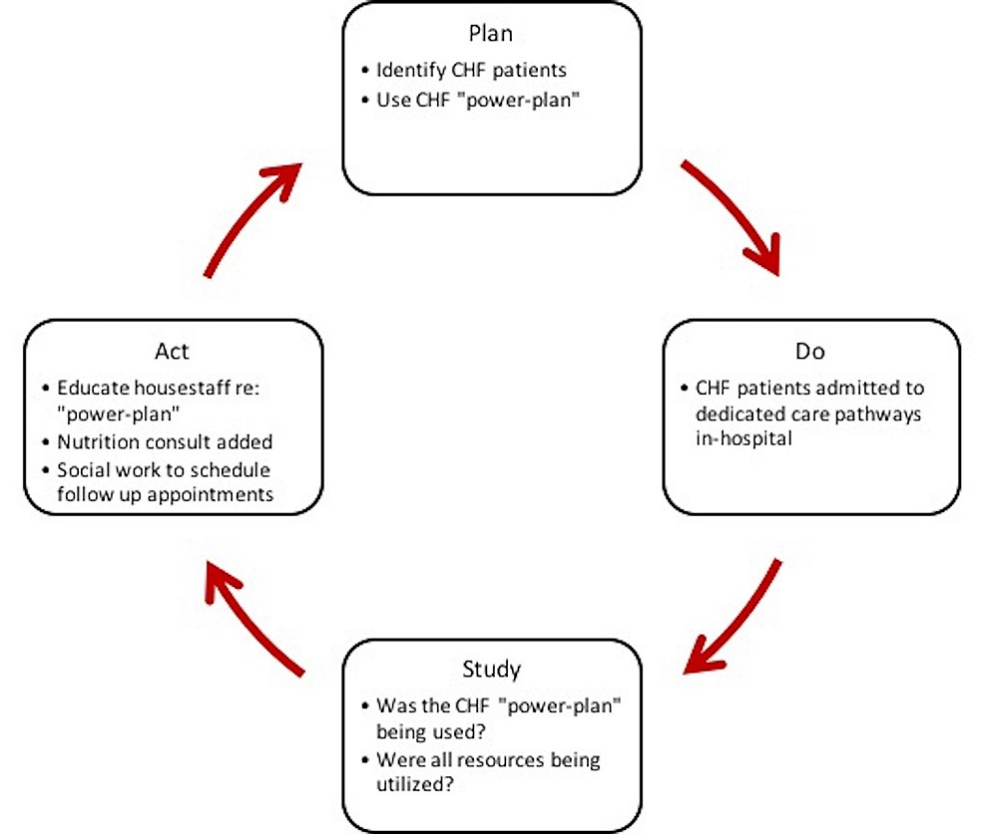
Plan-Do-Study-Act Cycle for implementation of a care coordination pathway CHF = Congestive heart failure

In order to implement our goals with the Care Coordination initiative, a 'Plan-Do-Study-Act' (PDSA) cycle was utilized [28]. Since our primary drivers were to optimize inpatient management of heart failure, we relied on two criteria to identify heart failure admissions: a) elevated NT-proBNP (N-terminal pro-B-type natriuretic peptide) greater than 300 and b) use of intravenous (IV) diuresis on admission. These clinical triggers would prompt the use of a dedicated “power-plan” in our hospital EMR which would include orders for intravenous or oral diuresis, inotropic or vasodilator support if indicated, but also an updated transthoracic echocardiogram, guideline-directed medical treatment [spironolactone, angiotensin-converting enzyme inhibitor (ACEi)/angiotensin receptor blockers (ARBs)/angiotensin receptor-neprilysin inhibitor (ARNI), diuretics, beta-blockers], daily weights, strict fluid input/output recordings, and a fluid-restricted diet. Moreover, consultation with dietary and pharmacy services was encouraged. At the start of implementation, an analysis via EMR found that the admissions “power-plan” was only being utilized in about 3% of indicated heart failure admissions; our institution was under-utilizing nutritionist education, pharmacist education, and scheduling of post-discharge appointments. These components drove the PDSA cycle for future iterations, as illustrated in Figure 1.

Input from the pharmacy, case management, and nutrition staff revealed the underutilization of these services during the heart failure patient's hospitalization. Orders for pharmacy, case management, and nutritionist consults were included in the admissions “power-plan” after the PDSA cycle. Case managers began paging residents to ensure the follow-up appointment was included in the discharge summary; pharmacists conducted medication reconciliation at both the times of admission and discharge; a nutritionist would meet with the patient at least once prior to discharge. 

Interdisciplinary rounds on the units were conducted to guide day-to-day management and address barriers, such as inaccurate intake/output recording or the need for home services. Our team developed a sustainable and standardized care pathway, and measured the utilization of pharmacy, nutrition, physical therapy, case management, and social work resources using the hospital EMR; outpatient follow-up appointments were made prior to discharge. These utilization rates were discussed weekly by the executive committee and prioritized among respective departments accordingly. We verified stakeholders among the case management nurses, resident physicians, clinical pharmacists, social workers, registered dieticians, home care nurses, and transitions of care coordinators to target interventions across the care continuum; it is summarized in Table 3.

**Table 3:** Stakeholders and interventions IV= Intravenous; IDR= Interdisciplinary rounds; SWA= Social worker assistant; DSRIP= Delivery system reform incentive payment

Stakeholders	Interventions
Clinical triggers	Pro Brain Natriuretic Peptide > 300, IV loop diuretics
Case Managers	Verify active heart failure patients during IDR
House Staff	Utilize “power-plan” with high-risk Heart Failure consult
Clinical Pharmacist	Reconciliation of medications on admission and discharge
Social Work/SWA	Deliver medications directly to beds
Registered Dieticians	Education completed on all heart failure patients
Volunteers	Discuss heart failure education booklets and make follow-up phone calls within 48 hours post-discharge
Home Care Nursing	Referrals for home nursing or social needs; supply scales
DSRIP goal	Schedule follow-up appointments to see a primary care physician/cardiologist within 7-14 days

We assessed the impact of our Care Coordination initiative by studying the prompt identification of heart failure patients, the percentage of heart failure patients for whom the EMR “power plan” was utilized, the utilization of nutritionist, cardiologist, and pharmacist resources, and the presence of a follow-up appointment made at discharge. The percentage of heart failure admissions that had a nutritionist consult note, cardiology consult note, or pharmacy medication reconciliation was measured by chart review. Utilization of the “power plan” was determined by the EMR, as was the presence of a follow-up appointment made at discharge. A pre-and post-implementation analysis of these measures was used to draw conclusions for this study.

## Results

**Figure 2:**
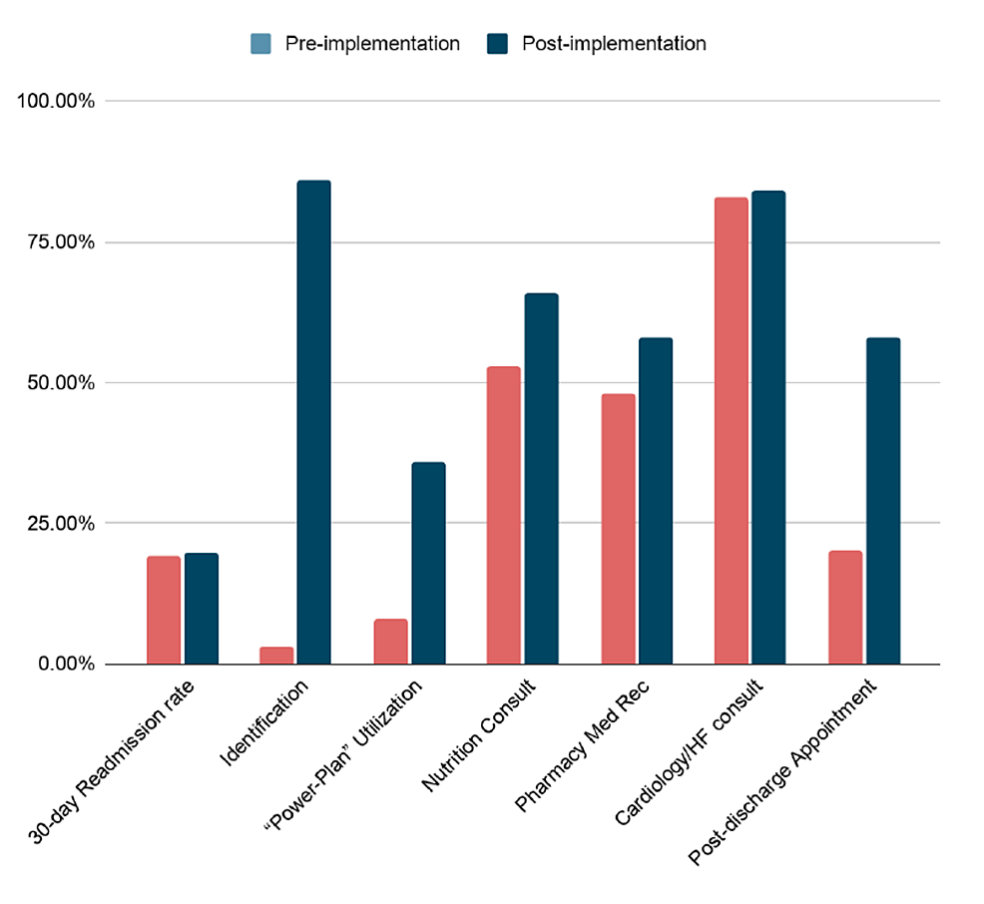
Pre-and post-implementation resource utilization rates Med Rec = Medication reconciliation; HF = Heart failure

After the implementation of our Care Coordination initiative, we began using the EMR to collect data on heart failure readmission rates, prompt identification of heart failure patients, utilization of the EMR “power plan”, presence of a nutritionist consult, pharmacist-driven medication reconciliation, cardiology consults, and primary care appointment made prior to discharge. These results are summarized in Figure 2 and Table 4. Patients were adequately identified 3% of the time prior to implementation and 86% of the time after implementation. The EMR “power plan” with relevant consults and acute-care best practices were used in 8% of relevant patients prior to implementation, and 36% of patients after implementation. Nutrition consults were placed in 53% of patients at the start of our intervention, and 66% of patients after our intervention. Pharmacist-driven medication reconciliation occurred in 48% of patients at the start of our intervention, and 58% of patients after our intervention. Involvement by cardiology/heart failure consultants did not change due to our intervention, occurring for 83% of our patients at the start of implementation and 84% after implementation. Post-discharge appointments nearly tripled, occurring for only 20% of our patients prior to implementation and 58% of patients after implementation. The 30-day readmission rate for heart failure did not markedly change from the implementation, 19.13% prior to implementation and 19.75% after implementation.

**Table 4:** Results NT-proBNP = N-terminal pro-B-type naturetic peptide; HF = Heart failure

Outcomes	Pre-implementation	Post-implementation
Heart failure 30-day Readmission rate	19.13%	19.75%
Identification with NT-proBNP, diuretics	3%	86%
Utilization of “Power-Plan”	8%	36%
Nutrition Consult	53%	66%
Medication Reconciliation by Pharmacy	48%	58%
Cardiology/HF consult	83%	84%
Post-discharge Appointments made	20%	58%

## Discussion

The management of heart failure patients starts in the hospital and continues in the community. Patients who are treated in a standardized, dedicated care pathway and managed by collective efforts of a multidisciplinary team have reduced morbidity and better outcomes. Identifying these patients early, involving a comprehensive team, and transitioning their care to the outpatient setting improves the quality of care in these patients.

The utilization of our “power plan” promoted adherence to a series of evidence-based best practices shown to improve outcomes in patients with heart failure. The involvement of the case management support team nearly tripled the number of outpatient appointments made for patients prior to discharge and aided in the transition of care from inpatient to outpatient management. Across all relevant hospital resources (nursing, nutritionist, pharmacy, cardiology) the use of a dedicated EMR “power plan” increased utilization. 

The aim of this Care Coordination initiative was not to decrease readmission rates but to appropriately identify patients in the acute-care setting and improve the transition of care to the outpatient setting. These interventions were aimed at incorporating best practices into the care of heart failure patients, but readmissions are challenging to address for a variety of reasons. The one-year mortality rate for heart failure patients in population-based studies is upwards of 35-40% [29,30]. Increased rates of readmission are associated with lower mortality rates in the heart failure population, and the introduction of readmission penalties in 2010 produced a paradoxical increase in 30-day and one-year mortality among heart failure patients [4,5]. 

Rather than focusing on reduced readmission, our aim was to focus on coordination of care, in the acute hospital setting as well as the transition to outpatient care. Poor medication compliance and socioeconomic/psychosocial factors account for a significant proportion of readmission risk [4,7,31]. A targeted approach towards care coordination across the inpatient and outpatient continuum optimizes patient outcomes, as well as high-quality discharge summaries and discharge follow-up appointments [32].

This study design has various strengths and limitations. The SQUIRE 2.0 standards for reporting quality improvement projects provide consistency in the way this non-randomized non-control tested project was implemented. We have before and after data, but this lacks the generalizability that a randomized controlled trial can. One of the major limitations of any study on heart failure readmission is determining if the readmission was a direct result of heart failure, or due to another cause. Many large studies use a consensus group to make that final adjudication, and our internal review of cases found that many readmissions were due to other patient comorbidities, such as sepsis or chronic obstructive pulmonary disease (COPD) exacerbation. 

For future interventions, we can identify patients with psychosocial/socioeconomic risk factors by formally assessing the cognitive status and investigating possible financial barriers to medication compliance. For example, delivering medications directly to the patient’s home might potentially remove another barrier to medication compliance. Also, regular assessment of health literacy in our heart failure patients using a validated questionnaire may help guide us towards the highest risk individuals. Finally, previous work demonstrates that high-quality discharge summaries reduce the risk of 30-day readmissions, particularly if the summary is transmitted to the patient’s primary care doctor [14,32]. Another initiative we have implemented is a phone call to the patient 48 hours post-discharge addressing heart failure symptoms, confirming the patient is on the correct medications and that the patient has a post-discharge follow-up appointment. 

## Conclusions

Overall, no single strategy has proven efficacious in a vacuum. Readmission penalties adversely impact safety-net hospitals and don’t take into account the social determinants of health that account for the large majority of poor health outcomes. A systems-wide approach engages everyone: doctors, nurses, pharmacists, nutritionists, case managers, and patients. Increasing access to each of these resources gives our patients the best chance to overcome the odds stacked against them, and our project serves as a model to emulate in other hospital systems.
